# The Epidemic of Mumps at Camp Wheeler

**Published:** 1919-01

**Authors:** 


***The Epidemic of Mumps at Camp Wheeler.*** By Lt. M. J. Radin. *The Archives of Internal Medicine*, Sept. 15, 1918.

*Etiology.* Smears from the region of the orifice of the duct in the cheek revealed a spirillum smaller and, in a few cases, more slender than the treponema pallidum. However, thus far the causative factor of mumps has not been determined. Experiments showed that the virus did not grow in ordinary mediums.

A factor of some importance was the previous condition of the men, who had been below par physically.

One attack does not confer immunity. Forty-seven patients gave a previous history of mumps, in the majority of which cases the gland on the side opposite to that of the present attack had been involved; but three patients had the same gland involved twice. One patient had mumps three times.

*Symptoms.* Often a diagnosis of mumps may be reached, before there is any swelling, from the following symptoms : (*a*) A feeling of stiffness in the jaws; (*b*) Difficulty in opening mouth; (*c*) Condiments,especially sour ones, cause a drawing sensation in the jaws; (*d*) Dry mouth may occur at first. No sense of taste, and no secretions. There may be salivation. Thirst at times unquenchable. (*e*) The orifices of Stenson's duct pout and a pinkish area appears around them; (*f*) The most diagnostic prodromal sign is tenderness just beyond the angle of the jaw. If the parotid gland is at all involved, the patient winces with pain. This occurs before any swelling can be made out.

*Onset.* (*a*) Onset with no symptoms; (*b*) Onset with no disturbances and features suggesting pancreatitis; (*c*) Onset with pancreatitis, orchitis and urethral discharge; (*d*) Onset with features of acute laryngitis and bronchitis; (*e*) Grippy onset with fever, heavy malaise, bone pains and aches, and sore throat; (*f*) The ordinary onset is with pain and swelling in the jaws, stiffness, difficulty in opening the mouth, slight malaise, and mild fever.

The course is variable. Special symptoms of all sorts arise. Elevation of temperature is not constant; however, fever occurred in over 80 % of the cases.

Vomiting, nausea, pain and tenderness in the testicles may occur without apparent cause.

Tenderness in the epigastrium, jaundice, and vomiting suggest pancreatitis.

Urinary retention may develop.

A patient may be doing well, apparently, and without cause develop the most marked nervous disturbances. Fainting, cold sweats, sense of collapse, diarrhea, an excessive irritability may occur. All grades of deafness have been present.

The uncomplicated cases show approximately a normal total leukocyte count (7,060). In orchitis, there is a definite leukocytosis (10,730) which is largely polymorphonuclear in character, that is, 6,470 polymorphonuclear cells per cubic millimeter, nearly double the number (3,660) in the uncomplicated cases; whereas the lymphocytes show a smaller increase, that is, 3,240 vs. 2,570 or a corresponding gain of one quarter.

The complications which may arise are orchitis, and epididymitis, otitis media, pancreatitis, pneumonia and bronchitis.

The following are diagnostic : (1) Hathcock's sign; (2) Pink and pouting orifices of Stenson's ducts; (3) Swelling and tenderness of the salivary glands; (4) Exudation of secretion from duct on pressure of gland; (5) Pain or peculiar drawing sensation on eating sour food.

In uncomplicated cases the infection subsided in a week. The average duration was fourteen days; limits, two to thirty-one days. Complications prolong the course about a week.

*Treatment.* A. *Prophylaxis.* (1) Careful isolation of cases for a period covering their infectivity — three weeks; (2) Some benefit resulted from five grains of hexamethylenamin four times daily for orchitis; (3) Two per cent. phenolized glycerin instituted routinely (2 drops into each nostril twice daily) has proved the most beneficial for otitis.

B. *Treatment Proper.* (1) Two compound cathartic pills on night of admission and saline in morning to insure free catharsis; (2) Dobell's gargle for mouth; (3) Hot application and camphorated oil to swelling; (4) For the pain and nervousness acetyl salicylic acid and bromide; (5) For fever, acetyl salicylic acid acetanilid; (6) phenol 2 per cent. in glycerin, two drops in each nostril twice daily; (7) Hexamethylenamin to reduce the incidence of orchitis, 5 grains four times daily; (8) For orchitis, support and counter irritation; ice bag; ten per cent. icthyol ointment; guaiacol carbonate; (9) Rest in bed, which is the best treatment.

